# Equity in health insurance coverage among women with children under two years of age in Laos: a cross-sectional study of sociodemographic disparities and implications for universal health coverage

**DOI:** 10.1186/s41043-026-01393-9

**Published:** 2026-07-20

**Authors:** Elizabeth Bunde, Kola A. Oyediran, Bouangern Xayalath, Jessica Posner, Nicole Davis, Souksakhone Inthavong, Banthida Komphasouk

**Affiliations:** 1 JSI Research & Training Institute, Inc., Saphantongtai Village, Sisatthanak District, Vientiane, Lao People’s Democratic Republic; 2 JSI Research & Training Institute, Inc., 2733 Crystal Drive 4th Floor, Washington, Arlington, D.C. Office, VA 22202 USA; 3 National Health Insurance Bureau, Ministry of Health, Simuang Road, Thatkhao Village, Sisatthanak District, Vientiane, Lao People’s Democratic Republic; 4 Independent Public Health Consultant, Vientiane, Lao People’s Democratic Republic

**Keywords:** Sociodemographic, Maternal health, Child health, Health insurance, Equity, Laos, Universal health coverage, Health financing, Interaction analysis

## Abstract

**Background:**

Achieving universal health coverage (UHC) in Laos requires a financing system that supports equitable access to essential services, especially for underserved groups. The National Health Insurance (NHI) scheme, integrating prior social and community-based programs, provides coverage for essential services and reduces out-of-pocket costs. Understanding socioeconomic factors shaping enrollment is critical to avoid policies that unintentionally reinforce inequities.

**Methods:**

We conducted a secondary analysis of cross-sectional survey data from 2,138 women with children aged 0–23 months across five Laos provinces. Multivariable logistic regression models estimated associations between sociodemographic factors and health insurance coverage, including an interaction term to examine provincial variations in the effect of education.

**Results:**

One-third of women had health insurance. Adjusted odds ratios (aORs) with 95% confidence intervals (CI) were estimated using multivariable logistic regression. Coverage was higher among older women, those with secondary education or above (aOR = 2.00, 95% CI: 1.57–2.54), employed women (aOR = 1.71, 95% CI: 1.30–2.24), women exposed to health messages (aOR = 2.03, 95% CI: 1.62–2.54), and those from wealthier households (highest vs. lowest quintile: aOR = 1.91, 95% CI: 1.23–2.91). Rural residence (aOR = 0.65, 95% CI: 0.48–0.87) and larger households (7–8 members: aOR = 0.68, 95% CI: 0.37–0.91) were associated with lower coverage. Provincial variation in maternal education showed Savannakhet and Oudomxay had relatively high coverage even among less educated women, whereas Phongsaly, Salavan, and Sekong displayed marked socioeconomic disparities.

**Conclusions:**

Health insurance enrollment is driven by a multidimensional set of factors, including education, household wealth, and geographic location. While provincial disparities and education are critical determinants, they interact with broader socioeconomic constraints. Achieving UHC coverage requires holistic interventions that not only target specific disadvantaged regions but also address the underlying social and financial barriers impeding access for vulnerable women.

## Background

Over the past two decades, Laos has made substantial progress in maternal and child health (MCH). Between 2017 and 2023, coverage of four antenatal care visits increased from 62.2% to 71.6%, skilled birth attendance rose from 64.4% to 79.8%, and postnatal care for newborns improved from 47.1% to 64.0% [1, 2]. Maternal mortality fell from 579 to 126 deaths per 100,000 live births between 2000 and 2020, and under-five mortality dropped by nearly 30%, to 43 per 1,000 live births by 2021 [3, 4]. These gains reflect strengthened health infrastructure, expanded financing, enhanced service delivery, and partnerships that have increased access to essential health services.

Despite these advancements, significant inequities remain. Access to quality care is uneven, particularly for women in rural areas, ethnic communities, and low-income households. Barriers such as cost, distance, transportation, perceived poor quality, and limited health literacy continue to impede service use [5]. These challenges are compounded by regional and economic disparities, with women in remote or disadvantaged areas facing limited facility access, shortages of trained personnel, and inadequate infrastructure [6, 7]. Evidence further suggests that poorly understood or insufficiently targeted health insurance schemes, combined with out-of-pocket payment requirements, may further reduce the utilization of essential services [8–10].

Although initiatives such as the Free MCH program expanded service coverage, poor, rural, and ethnic minority women continue to face substantial barriers to institutional delivery and essential MCH care, indicating uneven program reach [11]. These disparities are driven in part by socioeconomic stratification, which remains a strong determinant of access and reflects persistent structural inequities despite improvements in health financing and health system infrastructure [12]. To address these gaps, the National Health Insurance (NHI) scheme was launched in 2016, integrating Free MCH and other programs to enhance financial protection and broaden coverage for vulnerable populations [13, 14].

However, limited evidence exists on who enrolls in the NHI, whether enrollment is equitable across socioeconomic, geographic, and ethnic groups, or which individual or household characteristics shape enrollment decisions. Prior research on the Free MCH program and early NHI assessments suggest persistent inequities, but most studies have examined broader populations or service use rather than insurance coverage among mothers with children aged 0–23 months [11, 15]. Addressing this gap is critical, as this population is central to improving MCH outcomes across the continuum of care, including prenatal services, delivery, postpartum recovery, and early childhood immunization and illness management.

Healthcare in Laos is primarily delivered through the public system, with health centers providing primary care and district and provincial hospitals offering more advanced services. However, the system faces persistent constraints, including low public health spending, reliance on out-of-pocket payments, and critical shortages and maldistribution of the health workforce, which together constrain both access to and quality of care [16–18]. These constraints may also influence health insurance enrollment by shaping perceptions of service availability and reliability, particularly in rural areas [19–22].

To reduce financial barriers and promote equitable access, the NHI scheme covers outpatient and inpatient services, diagnostics, treatment, and high-cost interventions like hemodialysis [23, 24]. Vulnerable groups, including poor households, pregnant women, and children under five, receive additional support, such as transportation and food allowances during hospitalization [13, 25]. Co-payments and contributions vary by employment type. Formal sector workers contribute through salary-based payments (8% of salary, with 5.5% employer contribution) [14]. Informal workers pay modest co-payments (5,000 to 30,000 LAK for inpatient services and 5,000 to 20,000 LAK for outpatient care), with 50% of the premium subsidized by the government [26, 27]. Vulnerable groups are fully exempt [28]. Despite promotion through radio, television, and other media, awareness remains limited, with only about half of health facilities displaying NHI information [29].

Evidence from Southeast Asia underscores the important role of health insurance in improving MCH outcomes. Thailand’s universal health coverage (UHC) scheme, introduced in 2002 and funded through tax-based financing mode, significantly expanded access to healthcare, particularly for previously uninsured populations, including informal sector workers [30, 31]. Studies in Thailand and the Philippines show that insured women, especially in rural and low-income areas, are more likely to deliver in health facilities and use essential MCH services compared with uninsured women [32, 33]. Broader evidence indicates that insurance increases access to both outpatient and inpatient care, including maternal health services [34–36].

Despite these benefits, insurance coverage among women remains low in many low- and middle-income countries (LMICs). In Cambodia, only 24.9% of women report having health insurance, and a systematic review across 89 LMICs found an average coverage of 31.1%, including 27.3% in lower-middle-income countries [37, 38]. These gaps highlight the need to understand the determinants of enrollment, particularly in Laos, where research on women’s access to health coverage and its influence on MCH services use is limited.

**Fig. 1:**
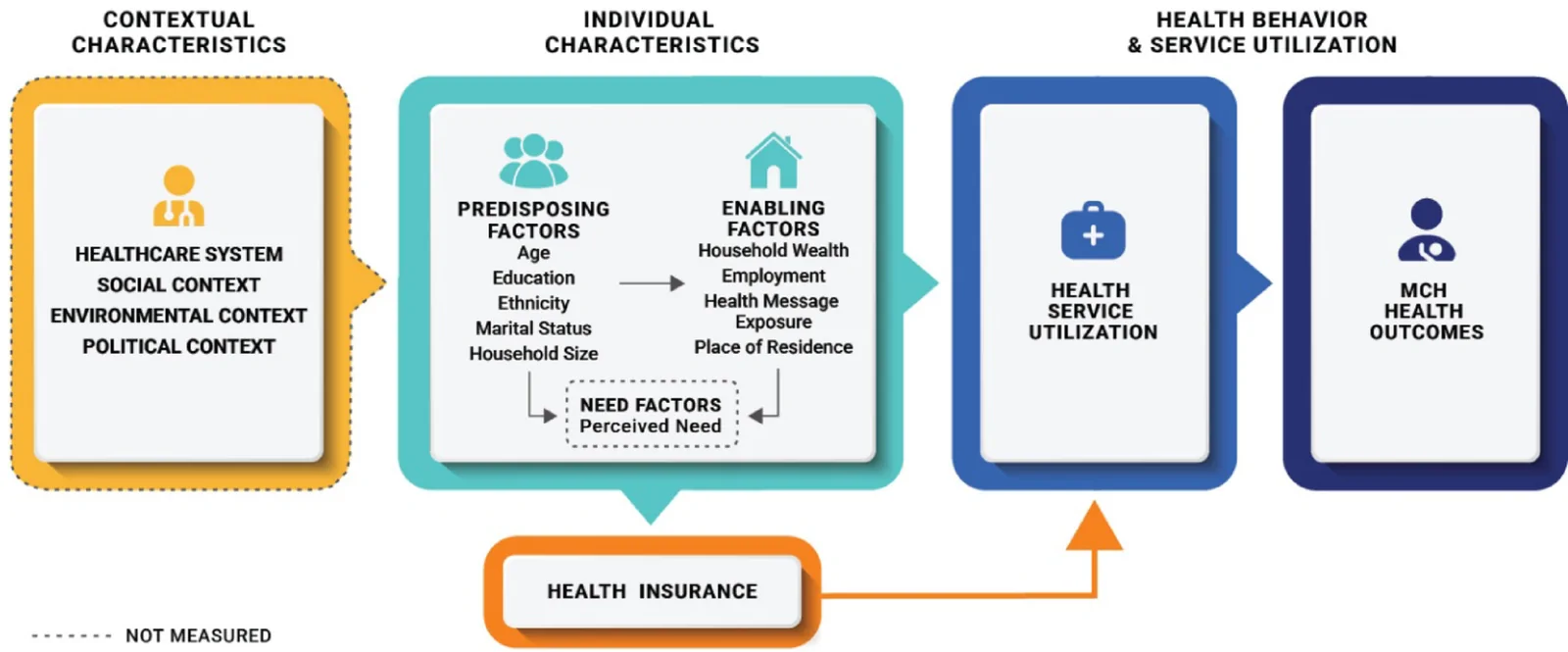
Conceptual framework of health care utilization, adapted fromAndersen's Behavioral Model

This study draws on Andersen’s Model of Health Care Utilization, which categorizes determinants of service use into predisposing, enabling, and need factors (Fig. 1) [39, 40]. Although need factors were not measured here, the selected predisposing and enabling variables align closely with the model. Empirical evidence from LMICs indicates that socioeconomic and demographic characteristics strongly influence insurance enrollment [41, 42]. A meta-analysis of 48 studies in 17 countries found that households in the highest wealth quintile were 61% more likely to enroll than the poorest, and individuals with the highest education levels were 64% more likely to enroll than those with the least education [43]. Country-level studies in rural Vietnam, for example, demonstrate that insurance knowledge, perceived premium costs, and socioeconomic status influenced enrollment decisions [44]. Other studies show that higher education enhances women’s ability to navigate insurance systems and understand coverage benefits [45, 46].

Contextual factors further shape these relationships. Geographic location and limited rural healthcare access can increase out-of-pocket expenditures and reduce the effectives of insurance [47]. Mass media exposure may also influence how education and wealth affect enrollment [48]. To capture these interactions, our study incorporates an education-by-province term, reflecting the expectation that education’s effect on enrollment varies across regions and different infrastructure, economic development, and healthcare access.

Limited evidence on the determinants of health insurance enrollment, particularly regarding predisposing and enabling factors across provinces, has constrained targeted policy development for equitable MCH access. Persistent structural and socioeconomic barriers continue to limit women’s access to insurance, despite regional expansions in coverage. While other Southeast Asian countries, such as Thailand and Cambodia, have examined enrollment patterns extensively, Laos remains under-researched, particularly for mothers with young children.

This study addresses these gaps by examining disparities in NHI enrollment across provinces and assessing the influence of predisposing and enabling factors using Andersen’s model. By identifying who enrolls, the study aims to inform policies and interventions that strengthen equitable access to care as Laos approaches its 2026 transition from least-developed country status.

## Methods

### Study setting

This research is a secondary analysis of baseline data from a larger, mixed-methods, cross-sectional study conducted under the United States Agency for International Development (USAID)-funded Laos Maternal Child Health Nutrition (LMCHN) project. This was a five-year initiative led by John Snow, Inc. (JSI) in partnership with the Lao Ministry of Health (MOH). LMCHN supported 24 districts across Phongsaly, Oudomxay, Savannakhet, Salavan, and Sekong provinces, strengthening over 230 health centers and district hospitals. The program aimed to increase access to high-quality MCH and nutrition services, build local health system capacity, and promote optimal health behaviors in communities.

The study area covers about 57% of the provincial districts and 59% of the population in the five provinces [49]. While these districts reflect the demographic and geographic diversity of the project area, they were not selected through nationally representative sampling. Accordingly, the findings are representative of the LMCHN area but should not be generalized to the provincial or national level.

Approximately 80% of the study provinces are rural (Table 1). Phongsaly, Sekong, and Oudomxay have much lower population densities − 11, 15, and 20 people per square kilometer – compared to the national average of 27, due to their mountainous geography. These provinces also experience high multidimensional poverty, with many communities reliant on subsistence farming [50]. While all the provinces are ethnically diverse, Savannakhet and Salavan are predominantly Lao-Tai (58% and 59%, respectively), whereas Phongsaly’s major ethnic group is Akha (32%), Oudomxay’s is Khmou (59%), and Sekong’s is Katu (23%).

**Table 1:** Characteristics of the assessed provinces

Province	Population^*^	Population density (population per square km) ^*^	% Rural ^*^	% Multidimensional poverty headcount rate ^† ‡^	% Major ethnic group ^*^	No. health facilities
Phongsaly	177,989	11	80.9	23.9	Akha: 32	39
Oudomxay	307,622	20	76.0	34.8	Khmou: 59	41
Savannakhet	969,697	45	77.8	33.9	Lao-Tai: 58	65
Salavan	396,942	37	88.7	32.9	Lao-Tai: 59	53
Sekong	113,048	15	64.8	44.1	Katu: 23	34

* 2015 Lao statistics bureau, population census

† 2018/19 Lao statistics bureau, poverty profile

‡ The population with a deprivation score of at least 33.3%, expressed as a share of the population

### Sample size and sampling design

The target population for this survey included households with women aged 15–49 years and a child aged 0–23 months. The sample size was calculated using Stata’s “power two proportions*”* command, based on five key MCH indicators: four or more ANC visits, skilled birth attendance, modern contraceptive prevalence, Diphtheria-Pertussis-Tetanus (DPT3) coverage, and full immunization. Baseline estimates (~ 50%) from the 2017 Lao Social Indicator Survey (rural areas) were used for conservative calculations [51]. The sample size was calculated to detect changes in key MCH indicators between the baseline and endline evaluations of the LMCHN project. Assuming a target increase from 50% to 62.5%, a one-sided chi-square test, α = 0.05, and 80% power, and a design effect of 1.5 to account for the cluster sampling design, the minimum required sample size was estimated at 2,040 households. Although the calculated was based on a pre-post comparison, this sample size provides sufficient power for the point estimates required in the present cross-sectional analysis.

The survey employed a cross-sectional, two-stage cluster design. In the first stage, villages were selected using population-weighted systematic random sampling stratified by province. In the second stage, 20 households per cluster were randomly selected for interview. As data on the number of eligible households were unavailable, enumerators worked with village authorities to compile lists of households with children 0–23 months for the sampling frame. To ensure adequate sample sizes, if a selected village contained fewer than 20 eligible households, it was combined with the nearest neighboring village to form a cluster with a sampling frame of at least 25 eligiblehouseholds. All eligible women in the selected households who consented to participate were interviewed.

A total of 111 of 1,425 villages (8%) were included, covering 23 of 24 districts, with 2,138 women interviewed, slightly exceeding the target by about 5%. This sample size was sufficient to detect program-relevant changes with 95% confidence and a 5% margin of error at both the provincial level and the study area.

### Data collection

The data were collected over an approximately five-week period in November and December 2022 from eligible women 15–49 years with children aged 0–23 months. The survey gathered household characteristics and assessed women’s knowledge, attitudes, and practices related to MCH. Eligible women were invited to participate at their homes. If a selected eligible woman declined or was unavailable, she was replaced by another eligible woman randomly selected woman from the household list.

Interviews were conducted in private settings, lasting 45–90 min.^1^ Most interviews were in Lao, but a few were conducted in local languages (Hmong and Khmu), with on-the-spot verbal translations provided by locally recruited data collectors who were fluent in both Lao and the local language. Pre-translated tools were available in English and Lao, but translations to Hmong and Khmu was not feasible, as these languages have no written form and finding qualified translators is challenging.

A total of 45 locally recruited data collectors were trained. Each province had a supervisor and 1–2 team leaders, depending on its size and geography. Additional oversight came from the MOH’s Department of Hygiene and Health Promotion (DHHP) and district and provincial authorities. Data were entered via tablets using the SurveyCTO platform, synched to a cloud-based platform, and reviewed daily for quality, completeness, and outliers.

### Variables

The outcome variable was health insurance coverage. Respondents were asked if they had coverage at the time of the survey and, if so, what type. A dichotomous variable was created (covered by any type of health insurance vs. not covered). This binary classification aggregates both mandatory schemes (social security) and voluntary or subsidized schemes (community-based insurance) to capture the overall status of financial protection among the target population, irrespective of the enrollment mechanism [52]. Respondents were classified as ‘uninsured’ if they reported no active coverage or lacked a valid membership card at the time of the survey. This distinction is critical because, although the NHI is theoretically universal, effective access to its low co-payment benefits often requires formal registration or possession of a membership card. Without which individuals may perceive themselves as lacking coverage or be subject to higher fee-for-service charges.

Independent variables were categorized based on Andersen’s model. Predisposing factors included maternal age (< 25 [reference], 25–29, 30–34, ≥ 35 years), education (primary or no education [reference] vs. secondary and above), ethnicity (Non-Lao-Tai [reference] vs. Lao-Tai), marital status (previously married [reference] vs. currently married/cohabiting), and household size (2–4 [reference], 5–6, 7–8, ≥ 9 members). Enabling factors included household wealth (divided into quintiles, with the poorest quintile as reference), employment status (unemployed, indicating no cash or in-kind payment for work inside or outside the home, either formal or informal [reference] vs. employed, indicating receiving cash or in-kind payment inside or outside the home), exposure to health messages (no, did not hear, see, or read any health messages related to family planning, child health, maternal or newborn health, or nutrition [reference] vs. yes), place of residence (urban [reference] vs. rural); and province (Phongsaly [reference], Savannakhet, Sekong, Salavan, and Oudomxay). In alignment with Andersen’s model, province and place of residence were conceptualized as contextual enabling factors that reflect the external health system environment and physical access constraints. An interaction term between education and province was also included.

Socioeconomic status was assessed using a household wealth index constructed via Principal Component Analysis (PCA) [53]. The analysis incorporated ownership of durable assets (television, refrigerator, mobile phone, motorcycle, car), housing characteristics (floor, roof, and wall materials), and utility access (drinking water source, sanitation facility, electricity). Categorical variables were dichotomized prior to inclusion. Using factor loadings from the first principal component as weights, continuous wealth scores were generated to stratify the sample into quintiles ranging from the poorest to the wealthiest.

To capture socioeconomic differences, education was treated as a binary variable, while household was categorized into quintiles to reflect the full range of socioeconomic status. This approach preserves meaningful distinctions in both variables and allows for the identification of groups at higher risk of under-enrollment.

Using Andersen’s model, variables were categorized as predisposing factors (e.g., age, education, ethnicity) and enabling factors (e.g., wealth, employment, residence), disentangling socioeconomic disparities while accounting for geographic and cultural barriers in Laos [54, 55]. In this framework, predisposing factors represent individual sociodemographic characteristics that influence the propensity to seek coverage, independent of specific health needs. Enabling factors reflect the financial and logistical resources that facilitate the actual ability to enroll. While Andersen’s model suggests that enabling resources may mediate the relationship between predisposing traits and utilization, this specific analysis focuses on assessing the independent associations of these factors to identify distinct barriers to insurance uptake.

Given the potential variability of predisposing and enabling factors across different regions, we also tested whether the effect of maternal education on NHI enrollment differed by province. This approach aligns with Andersen’s model, which suggests that individual determinants may be modified by local contexts, as seen in LMICs where socioeconomic disparities in insurance coverage depend on health system capacity and geographic factors.

### Statistical analysis

The analysis focused on 2,138 women (15–49 years) with complete data on all variables of interest. Less than 1% of the original sample was excluded due to missing values on key variables, resulting in the final analytical sample of 2,138. Statistical analysis was performed using Stata 17.0 [56]. Given the survey’s self-weighting design, no sampling weights were applied for estimating prevalence and odds ratios. To adjust for intra-cluster correlation, the vce(cluster) command was specified at the village level. This approach produces robust standard errors that account for the non-independence of observations within clusters. This approach ensures accurate statistical inference, with 95% confidence intervals and p-values reflecting the design effect from the multi-stage sampling strategy.

The prevalence of health insurance coverage was assessed through frequency analysis, while the relationships between health insurance coverage and sociodemographic variables were explored using univariate and multivariable logistic regression. Chi-square tests evaluated associations between sociodemographic factors and insurance coverage.

Multiple logistic regression models were used to calculate adjusted odds ratios (aOR) and 95% confidence intervals (CIs) for health insurance coverage. An interaction term was included to assess whether the effect of education on insurance enrollment differed by province. The regression model is shown below, with statistical significance set at *p* < 0.05. To validate the inclusion of the interaction term, a Likelihood Ratio Test (LRT) was conducted comparing the main effects model (Model 1) against the interaction model (Model 2). The test was statistically significant (X^2^ = 36.19, *p* < 0.001), confirming that the interaction model (Model 2) provided a better fit to the data.

Where:

- **Yi**: The binary outcome variable (1 = Insured, 0 = Uninsured).
- **Educi**: Maternal education level (categorical: No education [ref], Primary, Secondary+).
- **Provi**: Province of residence (categorical: Phongsaly [ref], Oudomxay, Savannakhet, Salavan, Sekong).
- **Educi × Provi**: The interaction term capturing the variation of education’s effect across provinces.
- **Z**_**ki**_: A vector of *k* control covariates categorized by Andersen’s model, including:
  - *Predisposing factors*: Maternal age, ethnicity, marital status, and household size.
  - *Enabling factors*: Household wealth quintile, employment status, exposure to health messages, and urban/rural residence.
- β and γ: The regression coefficients to be estimated.

This specification aligns with standard econometric frameworks for binary response models with interaction effects, as detailed in Wooldridge (2010) [57].

**Fig. 2:**
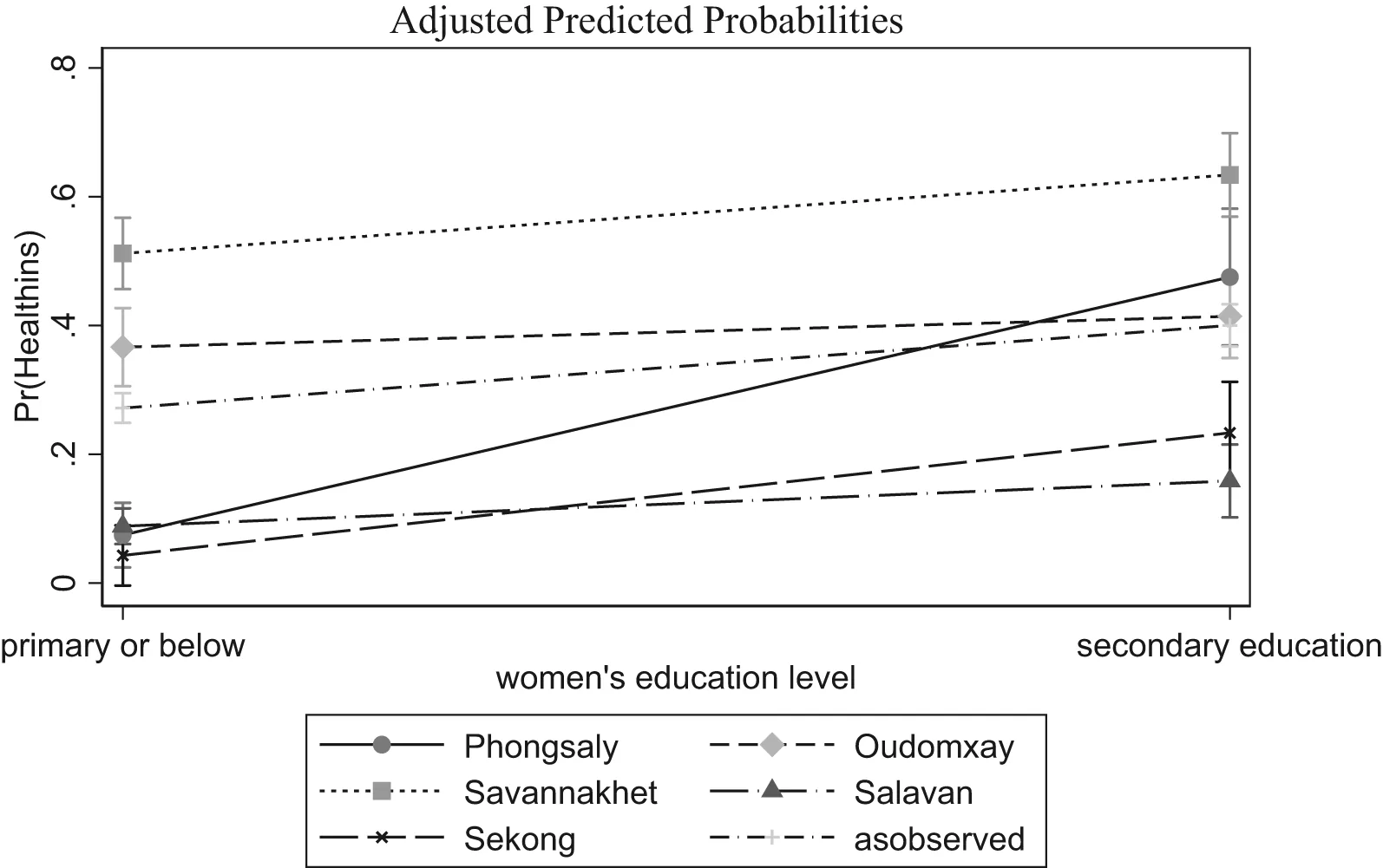
Adjusted predicted probabilities (with 95% confidence intervals) of health insurance coverage among women with children aged 0–23 months, stratified by education level and province of residence

If the interaction coefficient of β4 is significant, it would suggest that the association between education and the probability of women being insured varies by province of residence. Results are reported as aORs to maintain comparability with the public health literature. To enable interpretation, particularly regarding the interaction between education and geography, we also estimated adjusted predicted probabilities (Fig. 2). This approach provides absolute estimates of insurance coverage across sociodemographic profiles, complementing the relative associations captured by aORs. Odds ratios greater than one indicate a higher likelihood of health insurance coverage, while those less than one suggests a lower likelihood.

Multicollinearity among independent variables was assessed using the Variance Inflation Factor (VIF). In the main effects model (Model 1), the mean VIF was 1.86, and in Model 2, it was 2.99, with no individual variable exceeding the threshold of 5, indicating no concern for multicollinearity. Elevated VIF values in Model 2 were observed for education and province, However, this was attributed to structural multicollinearity from the interaction term, which does not affect the validity of the regression estimates.

### Ethical considerations

The study was approved by the National Ethics Committee for Health Research (NECHR) in Laos (Approval #077, 2022) and JSI’s institutional review board (Approval #23–51, 2022). All procedures involving human participants adhered to ethical standards set by the institutional and national research committees, as well as the 1964 Helsinki Declaration and its later amendments.

Participants provided verbal informed consent before participating, which was recorded in the tool. The consent process was conducted in Lao or, if necessary, the participant’s local language. The consent form explained the study’s objectives, requirements, potential risks, and privacy rights, emphasizing the voluntary nature of participation and the absence of negative consequences for refusal or withdrawal.

The study included only married women. Under local laws in Laos, married individuals under 18 are considered legal adults and may provide their own consent. All participants, regardless of age, voluntarily provided consent. Contact information was provided for any questions or concerns. The research team was trained in ethical practices and informed consent procedures.

### Funding

This research was funded by USAID through the LMCHN project, cooperative agreement number 72043921CA00001.

## Results

### Respondent profile

Among the 2,138 women interviewed, 32.7% had health insurance, obtained through direct payments or employment (Table 2). Of those insured, 66.2% were enrolled in community health insurance, 29.6% through social security, and the remainder through health equity insurance (2.2%), private health insurance (1.4%), or other means (2.2%). Health insurance coverage varied by province, with Savannakhet having the highest proportion of insured women (53.8%), followed by Oudomxay (41.4%), Phongsaly (23.2%), Sekong (16.9%), and Salavan (11.1%). Significant heterogeneity in insurance coverage was observed across socioeconomic and demographic groups in unadjusted analysis, with some variables showing significant associations with insurance status (*p* < 0.05).

**Table 2:** Socioeconomic and demographic characteristics of women with children aged 0–23 months (n = 2,138)

Characteristics	Frequency	Percent	Percent insured	P-value^1^
Health insurance coverage				
Insured	699	32.7		
Uninsured	1,439	67.3		
Women’s age				0.000
Below 25 years	808	37.8	23.3	
25–29 years	629	29.4	34.8	
30–34 years	440	20.6	39.8	
35 years and older	261	12.2	44.8	
Median age (IQR)	27 (9)			
Education level				0.000
Primary education or below	1,294	60.5	24.9	
Secondary education or above	844	39.5	44.7	
Marital union				0.082
Currently married or cohabiting	2,007	93.9	32.2	
Previously married	131	6.1	39.7	
Household wealth quintile				0.000
First wealth quintile	425	19.8	17.7	
Second wealth quintile	428	20.0	24.8	
Third wealth quintile	432	20.2	29.6	
Fourth wealth quintile	427	20.0	41.5	
Fifth wealth quintile	426	19.9	50.0	
Province of Residence				0.000
Phongsaly	254	11.9	23.2	
Oudomxay	505	23.6	41.4	
Savannakhet	627	29.3	53.8	
Salavan	568	26.6	11.1	
Sekong	184	8.6	16.9	
Place of residence				0.000
Urban	463	21.7	45.4	
Rural	1,675	78.3	29.2	
Ethnicity				0.000
Lao-Tai	1,167	54.6	37.9	
Non-Lao-Tai	971	45.4	26.5	
Religious affiliation				0.001
Buddhist	1,391	65.1	34.9	
Animist/Tai/folk/Satsana Phee	696	32.5	27.6	
Christianity/Islam/no-religion	51	2.4	41.2	
Employment status				0.000
Yes	1,670	78.1	34.7	
No	468	21.9	25.6	
Median number of people in household (IQR)	6 (2)			
Median pregnancy (IQR)	2 (2)			
Median number of children born alive (IQR)	2 (2)			

Numbers in parentheses are interquartile ranges (IQRs); LR = likelihood-ratio

^1^likelihood-ratio chi2 was used for the measure of association

### Factors associated with health insurance enrollment

The results of the logistic regression models are reported as odds ratios, with p values indicating the significance of the explanatory factors. Table 3 shows the predictors of health insurance enrollment among women in Laos.

The multivariable logistic regression revealed that women aged 25–29 years (aOR = 1.55; 95% CI: 1.19–2.03), 30–34 years (aOR = 2.03, 95% CI: 1.51–2.73), and 35 years and older (aOR = 2.66, 95% CI: 1.87–3.77) were more likely to have health insurance compared to women aged 24 or younger. Education was also a significant predictor, with women who had secondary education or higher (aOR = 2.00, 95% CI = 1.57–2.54) more likely to be enrolled than those with primary or no education.

Rural women were less likely to have insurance (aOR = 0.65, 95% CI: 0.48–0.87), while women in Savannakhet (aOR = 4.66, 95% CI: 2.95–7.37) and Oudomxay (aOR = 1.95, 95% CI:1.32–2.88) were more likely to be enrolled than women in Phongsaly. Conversely, women in Salavan (aOR = 0.38, 95% CI: 0.23–0.61) and Sekong (aOR = 0.46, 95% CI: 0.27–0.79) were less likely to be insured than were women in Phongsaly.

Exposure to health messages (aOR = 2.03, 95% CI: 1.62–2.54), employment (aOR = 1.71, 95% CI: 1.30–2.24), and higher household wealth were all associated with increased insurance enrollment. For example, women in the fifth wealth quintile (aOR = 1.91; 95% CI: 1.23–2.97) were 91% more likely to have insurance than were women in the first wealth quintile.

The analysis also revealed that household size had a nonlinear relationship with insurance enrollment, with women in households of 7–8 individuals (aOR = 0.68, 95% CI: 0.51–0.91) less likely to be insured than women in households with four or fewer people. Marital status and ethnic group affiliation were not significant predictors of enrollment.

**Table 3:** Bivariate and multivariable analyses of the factors associated with health insurance coverage among women with children aged 0–23 months

Explanatory factors	Unadjusted		Adjusted (model 1)		Adjusted (model 2) with interaction effect	
	Coef. (95%CI)	p value	Coef. (95%CI)	p value	Coef. (95%CI)	p value
Women’s age						
Below 25 years	Ref		Ref			
25–29 years	1.76 (1.40–2.22)	0.000	1.55 (1.19–2.03)	0.001	1.50 (1.15–1.96)	0.003
30–34 years	2.18 (1.69–2.80)	0.000	2.03 (1.51–2.73)	0.000	2.01 (1.49–2.71)	0.000
35 years and above	2.68 (1.99–3.59)	0.000	2.66 (1.87–3.77)	0.000	2.60 (1.82–3.70)	0.000
Education level						
Primary education or below	Ref		Ref			
Secondary education or above	2.44 (2.02–2.93)	0.000	2.00 (1.57–2.54)	0.000	13.44 (5.64–32.02)	0.000
Marital union						
Current in union	0.72 (0.50–1.04)	0.079	1.29 (0.85–1.95)	0.233	1.29 (0.85–1.95)	0.236
Previously married	Ref					
Household wealth quintile						
First wealth quintile	Ref					
Second wealth quintile	1.54 (1.10–2.14)	0.011	1.36 (0.94–1.97)	0.108	1.21 (0.83–1.77)	0.326
Third wealth quintile	1.96 (1.42–2.72)	0.000	1.32 (0.90–1.94)	0.156	1.15 (0.78–1.70)	0.486
Fourth wealth quintile	3.30 (2.41–4.53)	0.000	1.63 (1.08–2.43)	0.019	1.45 (0.96–2.18)	0.077
Fifth wealth quintile	4.67 (3.41–6.38)	0.000	1.91 (1.23–2.97)	0.004	1.74 (1.12–2.72)	0.014
Province of Residence						
Phongsaly	Ref					
Oudomxay	2.33 (1.66–3.28)	0.000	1.95(1.32–2.88)	0.001	8.20 (3.76–17.90)	0.000
Savannakhet	3.84 (2.76–5.35)	0.000	4.66 (2.95–7.37)	0.000	15.80 (7.07–35.34)	0.000
Salavan	0.41 (0.28–0.61)	0.000	0.38 (0.23–0.61)	0.000	1.21 (0.53–2.79)	0.653
Sekong	0.67 (0.41–1.09)	0.104	0.46 (0.27–0.79)	0.005	0.55 (0.14–2.17)	0.392
Place of residence						
Urban	Ref					
Rural	0.50 (0.40 −0.61)	0.000	0.65 (0.48–0.87)	0.004	0.70 (0.52–0.95)	0.022
Ethnicity						
Lao-Tai	1.69 (1.41–2.04)	0.000	0.88 (0.65–1.19)	0.406	0.93 (0.68–1.27)	0.646
Non-Lao-Tai	Ref					
Household size						
2–4 people	Ref		Ref			
5–6 people	1.05 (0.83 −1.33)	0.676	0.97 (0.74–1.27)	0.826	1.01 (0.77–1.32)	0.947
7–8 people	0.78 (0.61–1.01)	0.058	0.68 (0.51–0.91)	0.010	0.75 (0.56–1.01)	0.062
9 people or more	0.58 (0.40–0.84)	0.004	0.89 (0.58–1.37)	0.591	1.00 (0.64–1.55)	0.998
Exposure to health message						
Yes	1.33 (1.11–1.60)	0.002	2.03 (1.62–2.54)	0.000	2.08 (1.66–2.61)	0.000
No	Ref					
Employment status						
Yes	1.54 (1.22–1.94)	0.000	1.71 (1.30 −2.24)	0.000	1.63 (1.24–2.15)	0.000
No	Ref					
Education x province of residence						
Secondary education x Oudomxay					0.09 (0.04–0.23)	0.000
Secondary education x Savannakhet					0.13 (0.05–0.33)	0.000
Secondary education x Salavan					0.15 (0.05–0.41)	0.000
Secondary education x Sekong					0.55 (0.12–2.50)	0.442
LR Test (vs Model 1)					χ2 = 36.19	<0.000

Table 3 also presents the results of the model on the two-way interactionfactors between education and province of residence in the complete sample, adjusted for women’s sociodemographic characteristics and exposure to health messages. The multivariable logistic regression analysis revealed a statistically significant interaction between maternal education and province of residence (*p* < 0.05), demonstrating that the association between education and health insurance coverage is moderated by local geographic context.

As illustrated by the adjusted predicted probabilities in Fig. 2, the protective effect of education is significantly conditioned by location. Savannakhet (aOR = 4.66, 95% CI: 2.95–7.37) and Oudomxay (aOR = 1.95, 95% CI: 1.32–2.88) exhibit a high coverage “floor” where even uneducated women possess significantly greater odds of insurance than their counterparts in Phongsaly, Salavan, and Sekong. This suggests that systemic factors in these regions buffer against educational disadvantages. In contrast, Phongsaly displays a marked socioeconomic difference where access relies heavily on educational advancement, while Salavan (aOR = 0.38, 95% CI: 0.23–0.61) and Sekong (aOR = 0.46, 95% CI: 0.27–0.79) show persistently low coverage probabilities across all strata.

Crucially, significant interaction coefficients for secondary education in Oudomxay (aOR = 0.09) and Savannakhet (aOR = 0.13) suggest that higher education is generally associated with improved uptake. However, its relative advantage is markedly attenuated in provinces with stronger systemic support compared to the stark disparities observed in the reference province of Phongsaly.

In summary, older age, higher education, urban residence, employment, exposure to health messages, and greater household wealth significantly increased the likelihood of health insurance enrollment, while larger households decreased it. Provincial disparities were evident, with higher coverage in Savannakhet and Oudomxay and lower coverage in Salavan and Sekong. Education’s effect on enrollment was moderated by provincial context, highlighting regional difference in insurance uptake.

### Discussion

This study explored sociodemographic patterns in health insurance coverage among women with children under two years of age in selected provinces of Laos, with interpretation informed by Andersen’s Model of Health Care Utilization. The findings indicate that insurance enrollment is associated with a combination of predisposing factors (age, education), enabling resources (wealth, employment, exposure to health information), and contextual characteristics (province and place of residence). Importantly, the interaction between education and province indicates that individual-level resources translate into insurance coverage differently depending on local context. These findings should be interpreted as associations rather than causal relationships, given the cross-sectional design and the absence of direct measures of perceptions, preferences, or administrative processes.

### Predisposing factors

Age was positively associated with insurance coverage, with older women (aged 35 and above) significantly more likely to be insured than younger women (under 25). Within Andersen’s model, age is a predisposing factor reflecting both chronological and perceived life stage, which influences perceived need and patterns of health care utilization [58]. Older women are more likely to have had repeated interactions with health services through pregnancy, childbirth, or other health needs, increasing familiarity with insurance mechanisms and awareness of the financial implications of care [59–61]. In contrast, younger women may have fewer sustained interactions with the health system and may be more likely to experience unstable employment, an enabling resource within Andersen’s model that is strong associated with insurance enrollment, potentially constraining enrollment opportunities [62]. Although this study did not directly measure insurance literacy or perceived risk, age-related variation in coverage may reflect life-course differences in enabling resources rather than differences in motivation [63, 64].

Education was also associated with insurance coverage, with women who had secondary education or higher being more likely to be insured. Education is a core predisposing factor in Andersen’s model, influencing health-related knowledge, decision-making capacity, and the ability to navigate insurance systems [65–69]. However, this association varied by province. The significant interaction between education and province indicates that the relationship between education and insurance coverage differs across local contexts. As discussed further in the Contextual Interpretation section, disparities by education were more pronounced in some provinces than others.

Ethnicity was not a significant predictor of insurance coverage in the adjusted models. This contrasts with prior evidence documenting ethnic disparities in health access in Laos [70, 71]. One plausible explanation is that ethnic differences are indirectly captured through correlated structural factors such as province of residence, rurality, and household wealth, as several ethnic groups are geographically concentrated within specific provinces. The inclusion of province fixed effects may therefore attenuate the independent association between ethnicity and insurance coverage. Although this study did not formally estimate models excluding province effects, the findings suggest that ethnic disparities may operate primarily through geographic and structural pathways rather than as independent individual-level effects.

Marital status was also not significantly associated with insurance coverage. Given that the study population consisted exclusively of currently or previously married women with young children, marital status was limited. This may have reduced the ability to detect differences. In addition, marital status may influence insurance enrollment indirectly through household resources or employment, which were already included in the models.

Household size showed a nonlinear association with health insurance coverage, with women in household of seven to eight members less likely to be insured in the model without the interaction term. Within Andersen’s framework, larger household size may constrain enabling resources, as financial and caregiving resources must be distributed across more members [72]. This association may reflect resource dilution within larger households, although alternative explanations, such as household composition or extended family support, cannot be assessed here. After accounting for the interaction term, however, there was no association between insurance coverage and household size, suggesting that household composition alone may have less influence compared to broader contextual considerations.

### Enabling factors

In our analysis, household wealth was positively associated with health insurance enrollment, although the relationship was not strictly linear across quintiles. In the model without the interaction term, women in the two highest wealth quintiles were more likely to be insured than those in the poorest quintile. However, after including the education-province interaction, only women in the highest wealth quintile remained significantly more likely to be insured. This pattern suggests that enrollment is significantly higher among households in the highest (5th) wealth quintile, while no statistically significant differences are observed across the lower wealth quintiles.

These findings can be interpreted through Andersen’s model, in which wealth is commonly associated with fewer financial constraints on enrollment and health service use [73]. Although health insurance schemes in Laos are intended to limit out-of-pocket expenditures, indirect costs and co-payments may still restrict coverage among the most economically disadvantaged. Consistent with research from Laos and other LMICs, economic resources remain an important determinant of insurance coverage, particularly in systems that rely on partial contributions or voluntary enrollment [74–78].

Employment status was associated with insurance coverage, with women engaged in anypaid work more likely to be insured than those who were not employed, consistent with previous research in Laos [79]. Within Andersen’s model, employment functions as an enabling resource by providing access to income, social networks, and administrative systems that may facilitate enrollment. In this study, employment was measured broadly and did not distinguish between formal and informal work. Therefore, the observed association reflects differences between employed and non-employed women, rather than differences by employment type.

Exposure to health-related messages was positively associated with insurance coverage, with women who reported hearing seeing, or reading health-related messages more likely to be insured. Within Andersen’s model, access to information is considered an enabling factor that may support an individual’s ability to navigate health systems and administrative processes [80]. However, given the cross-sectional design, the direction of this association cannot be determined. Women who are already insured or engaged with health services may be more likely to encounter health messages through facility-based care or outreach activities. The association should therefore be interpreted as correlational rather than causal.

Place of residence remained a significant predictor of insurance coverage in the interaction model, with rural women less likely to be insured than urban women. This finding aligns with prior evidence highlighting geographic barriers to enrollment, including distance to facilities, limited administrative outreach, and weaker health infrastructure in rural areas [81, 82]. Although the magnitude of the association was reduced after accounting for the education-province interaction, rural residence retained an independent association with coverage, underscoring the continued relevance of geographic access with Andersen’s enabling domain.

### Contextual interpretation

The significant education–province interaction indicates that insurance enrollment reflects both individual characteristics and provincial context. In provinces such as Savannakhet and Oudomxay, we observed a high coverage ‘floor’ where even women with lower education levels had relatively high enrollment probabilities. This suggests that stronger local administrative capacity or community-based safety nets in these areas may compensate for lower individual health literacy. Conversely, in Phongsaly, a steep socioeconomic gradient was observed, indicating that without systemic support, access to insurance relies heavily on an individual’s own educational attainment.

These findings align with Andersen’s framework, suggesting that ‘contextual enabling factors’ - such as healthcare infrastructure and local governance - can modify the influence of individual predisposing factors. In regions with limited economic development or administrative outreach (e.g., Salavan and Sekong), neither education nor existing safety nets appear sufficiently consistent associated with higher enrollment, resulting in persistently low coverage across all groups.

This pattern mirrors experiences in neighboring Thailand, where decades of investment in district-level infrastructure and strong local governance were critical to reducing geographic disparities and achieving UHC [83]. Similarly, Cambodia has sought to protect the poorest through targeted financial mechanisms such as the Health Equity Fund [84]. While these comparisons are illustrative rather than directly comparable, they highlight how sustained subnational investment and targeted financial protection mechanisms can influence enrollment patterns. In contrast, Laos operates a fragmented set of insurance schemes with uneven provincial implementation and limited fiscal space, contributing to persistent geographic and socioeconomic inequities in coverage [85, 86]. These examples emphasize that provincial context matters for understanding enrollment disparities in Laos.

### Policy and program implications

The following implications are offered as considerations for policy discussion informed by observed enrollment patterns. First, the strong association between household wealth and enrollment, even within a system designed to be low-cost, suggests that current premiums or indirect costs remain a barrier for the near-poor. Policy adjustments, such as flexible installment payments or expanded subsidization for lower-wealth households, could help address financial barriers to enrollment. Second, the independent negative association between rural residence and coverage highlights the need to decouple insurance access from physical infrastructure. Expanding mobile enrollment units and leveraging village health workers for outreach could help overcome the geographic barriers identified in this study.

Finally, the marked provincial disparities indicate that a ‘one-size-fits-all’ national strategy is insufficient. Resources should be disproportionately targeted toward ‘low-floor’ provinces like Salavan and Sekong, where structural barriers appear to inhibit enrollment regardless of a woman’s education level. Conversely, in provinces with marked socioeconomic disparities like Phongsaly, interventions should prioritize simplified communication strategies to reach less educated women who are currently being left behind.

### Limitations

This study has several limitations. First, data were collected only in the LMCHN project’s operational area and, except for Sekong, did not cover all districts within each province. Therefore, findings are not fully representative of the provinces or of Laos as a whole. The focus on women with children aged 0–23 months further limits generalizability to other groups, including informal workers, men, older adults, or women without young children. Future studies should include a broader population to capture more comprehensive coverage patterns and inform equitable policy decisions.

Second, measurement and reporting issues may have affected response accuracy. Misunderstandings of health insurance terminology, as observed in previous studies, could introduce bias. Self-reported data are also subject to recall and social desirability effects, particularly for sensitive variables such as income, employment, and insurance enrollment. While the study employed a self-weighting design, sampling weights were not recalculated to adjust for non-response or potential inaccuracies in the village population data, which may further limit representativeness.

Third, the cross-sectional design limits causal inference, and results may be influenced by omitted variables and unmeasured confounding, including distance to health facilities or urban centers, which were not directly measured. Although models adjusted for urban-rural residence, this binary indicator may not capture variation in geographic access, including travel time and connectivity. Women who self-reported as pregnant were excluded, limiting the applicability of findings to this group and highlighting an important area for future research.

## Conclusion

Improving MCH outcomes requires attention not only to technical interventions but also to the social and contextual factors that shape health-seeking behavior. Expanding health insurance coverage for women of childbearing age is an important mechanism for improving access to MCH services and promoting equitable health outcomes. This study highlights key factors, both predisposing and enabling, that influence insurance enrollment, underscoring the importance of targeting under-resourced and underserved populations. Efforts to expand coverage may benefit from addressing financial, structural, and geographic barriers – including investments in infrastructure, improved outreach, and flexible premium models – and from approaches that account for regional variation in health system context. These strategies are essential for advancing UHC, reducing health disparities, and driving lasting improvements in MCH outcomes.

We are grateful to the Ministry of Health (MOH) and Lao People’s Democratic Republic for their support and for allowing us to conduct the assessment, especially the Department of Hygiene and Health Promotion (DHHP). We also thank the health management teams and facility staff at the provincial and district offices for supporting our data collection teams and data collection processes.

## Data Availability

The datasets generated and/or analyzed for the current study are available in the Figshare repository (DOI: [https://doi.org/10.6084/m9.figshare.29505173.v1]). The data will be made publicly available after the publication of a related manuscript. The datasets generated and/or analyzed for the current study can also be made available from the corresponding author upon reasonable request.Data Citation: Bunde, Elizabeth (2025). KAP Baseline Study Laos Maternal Child Health and Nutrition Project. figshare. Dataset. [https://doi.org/10.6084/m9.figshare.29505173.v1]).

## Abbreviations

aOR: Adjusted odds ratio
CI: Confidence interval
DHHP: Department of hygiene and health Ppromotion
DPT3: Diphtheria-pertussis-tetanus vaccine (third dose)
JSI: John Snow, Inc
LAK: Lao kip
LMCHN: Laos maternal child health and nutrition
LMIC: Low-and-middle-income countries
LRT: Likelihood ratio test
MCH: Maternal child health
MOH: Ministry of health
NECHR: National ethics committee for healthresearch
NHI: National health insurance
PCA: Principal component analysis
UHC: Universal health coverage
USAID: United states agency for international development
VIF: Variance inflation factor
WHO: World health organization

## Footnotes

1 The variation in interview duration (45–90 min) was primarily due to the need for on-the-spot translation in non-Lao-speaking households. Additionally, interruptions were occasionally necessary to maintain confidentiality when third parties entered the interview space, a common challenge in home-based surveys.
